# The Role of FeCoNiCrAl Particle Pretreatment in Interface Bonding and Properties of Cu/FeCoNiCrAl Composites

**DOI:** 10.3390/ma19030472

**Published:** 2026-01-24

**Authors:** Rui Zhu, Shaohao Zong, Xinyan Li, Jiacheng Feng, Wenbiao Gong

**Affiliations:** 1 School of Mechanical and Vehicle Engineering, Bengbu University, Bengbu 233030, China; 2 Key Laboratory of Advanced Structural Materials, Ministry of Education and School of Materials Science and Engineering, Changchun University of Technology, Changchun 130012, China

**Keywords:** diffusion layer, high-entropy alloy, Cu-matrix composites, friction stir processing

## Abstract

When fabricating high-entropy alloy particle-reinforced metal matrix composites via friction stir processing, the relatively low heat input led to insufficient interfacial diffusion between the particles and matrix, thereby compromising the composite properties. To address this issue, this study introduced an electroless copper plating step followed by heat treatment to produce Cu-coated HEA particles with an interfacial diffusion layer. These modified particles were then incorporated into a copper matrix via friction stir processing to form composites with an intentionally designed interfacial diffusion layer. The results indicate that the diffusion layer structure contributed to excellent interfacial bonding. The resulting composite exhibited a simultaneous enhancement in both strength and ductility. The tensile strength and elongation reached 372.5 MPa and 34.2%, respectively, representing increases of 20.4% and 54% compared to pure copper. The wear rate of the composite reduced by 33.7% relative to pure copper. Quantitative analysis indicated that the contribution of fine-grain strengthening, Orowan strengthening, dislocation strengthening, and load transfer strengthening to the overall strength was 41.2 MPa, 0.3 MPa, 12.7 MPa, and 15.7 MPa, respectively.

## 1. Introduction

Copper and its alloys were extensively employed as structural materials in aerospace, marine transportation, and related engineering fields owing to their exceptional combination of plasticity, thermal/electrical conductivity, and corrosion resistance properties. However, the strength of existing copper-based materials was limited, and it was difficult to further enhance them through conventional strengthening techniques, resulting in a gradual decline in their competitiveness within high-end equipment manufacturing applications.

The incorporation of reinforcements into metallic matrices to fabricate advanced composite materials with enhanced properties has been extensively investigated in recent years [1,2,3,4]. The types of reinforcement exerted a significant influence on the properties of the base material. Currently, ceramic particles, particularly oxides [5,6], nitrides [7], and carbides [8,9,10,11], are commonly employed as reinforcement. The incorporation of ceramic particles enhanced the strength of the metal, but considerably sacrificed its plasticity, ultimately restricting the application of the composite material. Thankachan [12] prepared copper surface composites reinforced by BN particles with volume fractions of 5%, 10%, and 15%, respectively. When BN particle content was 15%, the tensile strength of the composite reached 165 MPa. However, its elongation decreased dramatically (10.32%), which was only 53.2% of that of pure Cu (19.4%). Ostovan [5] fabricated Al5083-based composites with Al_2_O_3_ and Al-Al_2_O_3_ particles as reinforcement, respectively. The results showed that the mechanical properties of both composite materials were improved. But as compared with Al5083 alloy, their elongation was decreased by 80% and 64%, respectively. To address the issue of plasticity reduction of composites, some researchers substitute rigid metal particles for ceramic ones as reinforcement. However, due to the limited inherent strength of the rigid metal particles themselves, their strengthening effect on the matrix was not significant [13]. In summary, it was necessary to explore new materials as reinforcement to promote the synergistic increase of strength and plasticity of the matrix in composite materials.

The unique multi-principal-element design endowed high-entropy alloys (HEAs) with remarkable strength-ductility synergy and thermal stability, driving substantial research focus. Some scholars have successfully fabricated metal matrix composites reinforced with HEAs. Chen [14] fabricated HEA/Cu matrix composites (CMCs) via powder metallurgy, reporting yield strengths of 240 MPa (10 wt% HEA) and 330 MPa (20 wt% HEA). These findings suggest that HEA exhibited superior reinforcing effects in particle-reinforced CMCs compared to metallic glass. Li [15] prepared HEA-reinforced aluminum matrix composites (AMCs) at various sintering temperatures. At 480 °C, the composite achieved a compressive strength of 421.2 MPa, coupled with a peak shrinkage rate of more than 65%, demonstrating superior strength and ductility.

As a novel solid-phase processing technology, friction stir processing (FSP) has also been employed to fabricate metal matrix composites (MMCs). This technology facilitated grain refinement of the matrix and consequently enhanced the material’s mechanical properties [16,17]. Han [18] created CoCrFeNi HEA-reinforced Al matrix composites (AMC) via cold spraying and subsequently processed the composite using FSP. The mechanical properties of the FSP-processed samples were all higher than those of the CS-processed samples. Huang [19] prepared AMC reinforced with SiC particles by a combined method of PM and FSP. The grain size of the composite was only 2.06 μm. The yield strength and elongation of the nanocomposites prepared by friction stir processing (FSPed nanocomposites) were 75% and 164% higher than those of as-PM nanocomposites, respectively. This synergistic effect of improving strength and ductility indicated that FSP was a promising method for strengthening composite materials.

Interface bonding ability was also an important factor affecting material properties [20]. Yu [21] prepared Al_0.3_CoCrFeNi HEA particle reinforced CMC with an interfacial layer structure. The transition layer width between HEA and Cu was about 5 μm. The transition layer led to a 30% increase in the wear resistance of the composite. Yuan [22] fabricated CoCrFeNiMo_0.2_ HEA/Ti composites, revealing that the interface layer characteristics critically governed the composite properties. Within the 700–850 °C range, the mechanical properties of the composite exhibited a positive correlation with the interface layer width. Beyond the optimal thickness, however, property degradation occurred. Liu [23] demonstrated that sintering temperatures exceeded 560 °C triggered the formation of a single face-centered cubic (FCC) phase at the interfaces. An appropriate amount of FCC phase was conducive to improving the mechanical properties of the composite.

The small heat input during FSP due to the characteristics of solid phase processing, coupled with the slow diffusion effect of high-entropy alloys [24], results in weak interfacial diffusion ability between HEA and matrix. Therefore, improving the interface bonding ability was the key to creating HEA/Cu composites by FSP. Usually, the prepared composites were subjected to heat treatment to address these issues. However, this method could have a negative impact on the metal matrix (such as oxidation [25] and grain growth of the copper matrix, which led to a decrease in the performance of the composite materials). In this paper, the HEA particle was treated with electroless copper plating, resulting in HEA coated by copper (Cu@HEA). Then the Cu@HEA was heat-treated with a vacuum heat treatment furnace. The Cu@HEA after heat treatment formed a diffusion layer at the interface. These particles, after heat treatment, were used as reinforcement to prepare CMC by FSP. The aim was to improve the interfacial binding ability of HEA and Cu matrix, while avoiding the negative effects of heat treatment on the matrix.

## 2. Materials and Methods

**Figure 1:**
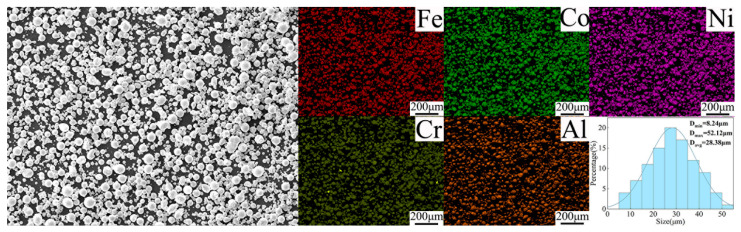
Morphology of FeCoNiCrAl HEA [26].

Pure copper (dimensions: 300 mm × 80 mm × 4 mm) served as the matrix material (Produced by Taizhou Biling Metal Products Co., Ltd., Taizhou, China). The reinforcement used was FeCoNiCrAl HEA (Produced by Chengdu Ketaolong Alloy Co., Ltd., Chengdu, China) after pretreatment (electroless copper plating + heat treatment). Figure 1 shows the microscopic morphology of the particle. Its element content was shown in Table 1. In a previous study, Cu matrix composites were successfully prepared with FeCoNiCrAl HEA as reinforcement via FSP (FSPed HEA/Cu) [26]. In order to facilitate comparison, the relevant parameters of pure copper and FSPed HEA/Cu were listed in Table 2.

**Figure 2:**
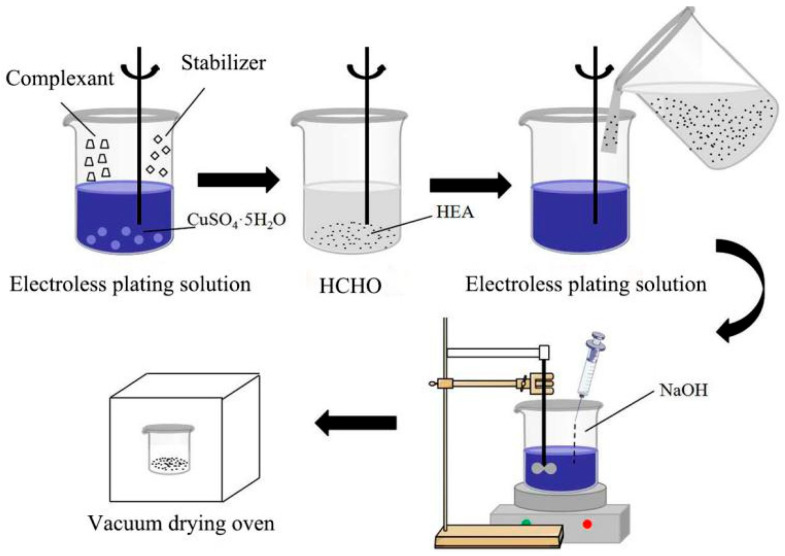
Technical process of electroless copper plating.

Figure 2 illustrates the electroless copper plating process, while Table 3 summarizes the corresponding plating bath composition and reaction temperature. Before electroless plating, FeCoNiCrAl HEA particles were pickled with concentrated sulfuric acid solution for 30 min and then washed with deionized water. The specific electroless plating process was as follows: (i) CuSO_4_·5H_2_O and KNaC_4_H4O_6_·5H_2_O were mixed to form electroless plating solution. (ii) We added a certain amount of HEA particles into the HCHO solution until the surface of HEA particles was thoroughly wet, and then poured this into the electroless plating solution. (iii) We stirred the mixed solution with a mixer at 50 °C, and dropped the NaOH solution into the mixed solution to adjust the PH value above 12. (iv) After the chemical reaction, the precipitate was separated from the solution and washed with deionized water. (v) The precipitate was subjected to vacuum drying, producing the desired Cu-coated HEA particles (Cu@HEA).

**Figure 3:**
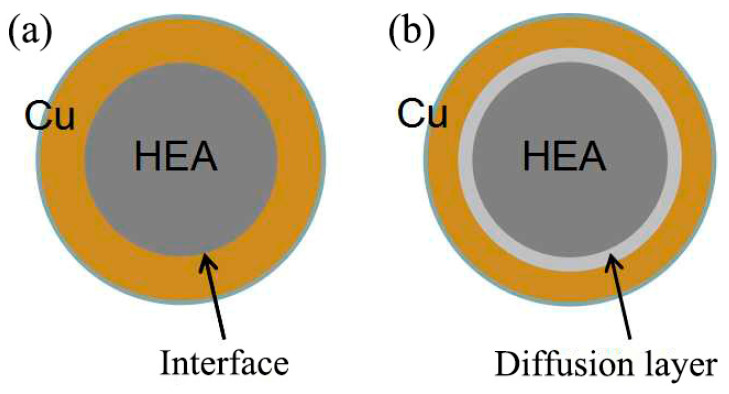
Cu@HEA particles (**a**) without heat treatment (Cu@HEA); (**b**) with heat treatment (Cu@HEA-DL).

The prepared Cu@HEA particles were held at 800 °C for 30 min in a vacuum heat treatment furnace, Cu@HEA particles with a diffusion layer structure (Cu@HEA-DL) were obtained, as illustrated in Figure 3. The Cu@HEA-DL particles served as reinforcement to fabricate composites with a distinct interfacial diffusion layer via FSP (FSPed Cu@HEA/Cu-DL). For comparison, composites without a pronounced diffusion layer (FSPed Cu@HEA/Cu) were simultaneously prepared using the non-heat-treated Cu@HEA particles under identical FSP conditions.

**Figure 4:**
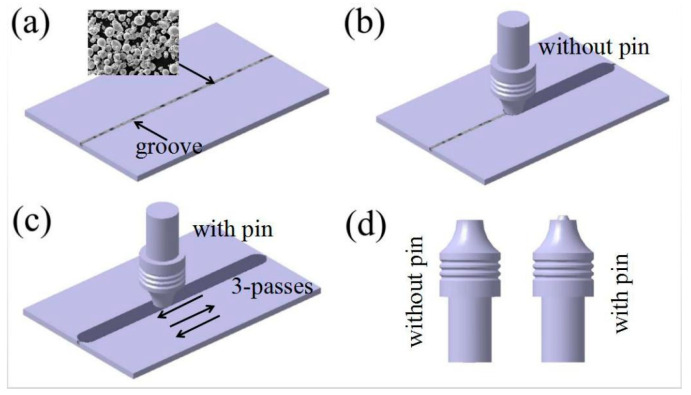
Schematic diagram of composite prepared via FSP [26]. (**a**) add particles; (**b**) Closed processing; (**c**) FSP processing; (**d**) tools.

Figure 4 illustrates the process of FSP to prepare a composite. Initially, a groove measuring 1.5 mm in width and 2 mm in depth was machined into the matrix and subsequently filled with reinforcement. A pinless tool with a shoulder diameter of 12 mm was then employed to seal the groove. Finally, three FSP passes were performed along the groove using a tool equipped with a pin (pin length: 2 mm). The LM3324-2D-13-T FSP machine was manufactured by Beijing FSW Technology Co., Ltd. (Beijing, China).

The microstructure and phase composition of the composites were characterized by X-ray diffractometer (XRD, Bruker–D8 ADVANCE, Bruker, Karlsruhe, Germany), scanning electron microscopy (SEM, Gemini Supra 40, ZEISS, Baden–Württem, Germany), energy dispersive X-ray spectroscopy (EDS), backscattered electron diffraction (EBSD), and transmission electron microscopy (Talos-FEI TEM, Thermo Fisher, Waltham, MA, USA). The microhardness was measured with the Vickers microhardness tester (Hengyi–EM500, Hengyi, Shanghai, China) under a 200 N load and a 15 s dwell time. The nanomechanical properties across the HEA particle-Cu matrix interface were evaluated via nanoindentation (Agilent G200, Agilent, Santa Clara, CA, USA) at a constant load of 30 mN. The tensile tests were conducted on a universal testing machine (WDW-200, Guanteng, Changchun, China) with a strain rate of 1 × 10^−3^/s, while tribological behavior was assessed using an reciprocating tribometer (RECT–MFT5000, Rtec–Instruments, San Jose, CA, USA) under a 30 N normal load, 5 Hz frequency, and 600 s test duration. All the tests were conducted three times.

## 3. Results

### 3.1. Morphology of Reinforcement

**Figure 5:**
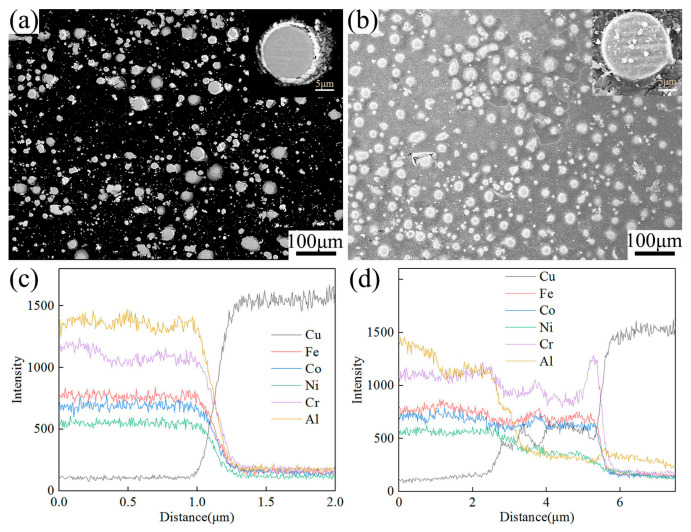
The SEM results of reinforcement (**a**,**c**) Cu@HEA; (**b**,**d**) Cu@HEA-DL.

Figure 5 shows the SEM image of the cross-section of the reinforcement. From the figure, an obvious Cu coating layer was seen around the surface of the HEA particles, suggesting a successful reaction of electroless copper plating. Interfacial voids were evident between the coating and matrix in the Cu@HEA particles (Figure 5a). In contrast, the Cu@HEA-DL particles exhibited improved bonding, with no visible voids (Figure 5b). Figure 5c further revealed negligible elemental interdiffusion in Cu@HEA, whereas a continuous diffusion layer approximately 3 μm thick formed in Cu@HEA-DL (Figure 5d). Similar investigations have also observed this diffusion phenomenon. This diffusion phenomenon also existed in similar investigations [23,27,28].

### 3.2. Interface Structure of Composite

**Figure 6:**
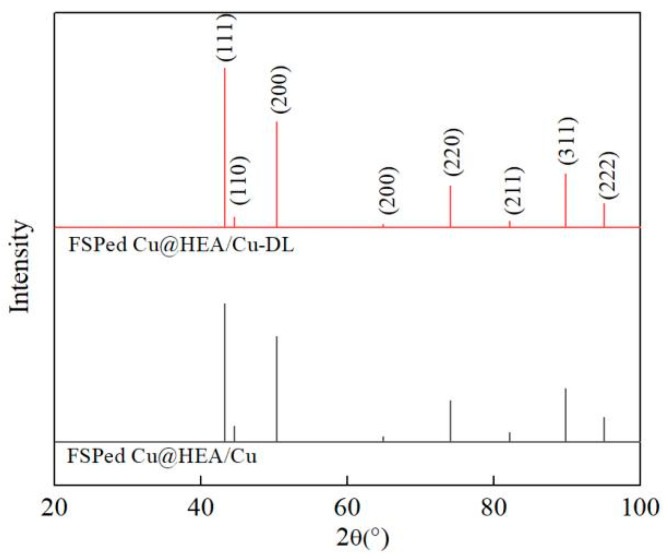
The XRD pattern.

Figure 6 presents the XRD patterns of both composites. Both patterns exhibited diffraction peaks corresponding to body-centered cubic (BCC) and face-centered cubic (FCC) phases, consistent with the initial HEA (BCC) and pure Cu (FCC) structures [26]. No obvious intermetallic compounds were observed. The undetected diffusion layer structure may be related to its small content, and further analysis by SEM and TEM is required.

**Figure 7:**
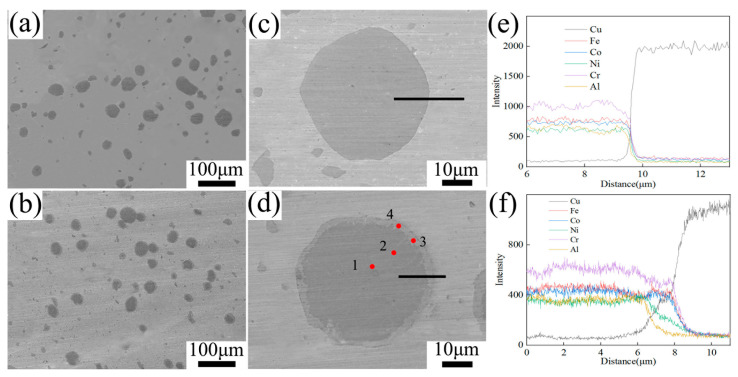
The SEM results (**a**,**c**,**e**) FSPed Cu@HEA/Cu; (**b**,**d**,**f**) FSPed Cu@HEA/Cu-DL.

Figure 7 displayed the SEM images of the composites. The FSPed Cu@HEA/Cu showed no discernible interfacial diffusion layer. In contrast, a well-defined diffusion layer was observed in FSPed Cu@HEA/Cu-DL, consistent with the prior observations of the reinforcement. By increasing the magnification of the SEM image, it was found that the diffusion layer region of FSPed Cu@HEA/Cu-DL had two phases, black and gray (Figure 7d). Line scanning was performed on the interface, and the results were shown in Figure 7e,f. The interface widths of FSPed Cu@HEA/Cu-DL were 3.5 μm. This result was slightly higher than that of Cu@HEA-DL, primarily because the effect of the heat generated during FSP on elemental diffusion cannot be ignored.

The EDS results of each point in Figure 7d were displayed in Table 4. The elemental composition of points 1 and 2 was discovered to be comparable to that of the original HEA particles, suggesting that the internal structure of HEA particles was unaffected by the diffusion heat treatment procedure and the ensuing friction stir processing procedure. The presence of Cu elements at both point 3 and point 4 in the diffusion layer region further proves the occurrence of diffusion behavior in this region. According to Equations (1)–(4) [18], it can be calculated that ΔHmix, ΔSmix, δ (atomic radius difference), and VEC (electronegative difference) at point 3 were respectively −0.308 kJ/mol, 13.611 J/k·mol, 3.169, 8.316, and ΔHmix, ΔSmix, δ, and VEC at point 4 were respectively 4.914 kJ/mol, 13.602 J/k·mol, 2.902, 8.971.

(1) $${\Delta H}_{\mathrm{mix}}=\sum_{i=1,i\neq j}^{n}\Omega_{ij}C_{i}C_{j}$$

(2) $${\Delta S}_{\mathrm{mix}}=-R\sum_{i=1}^{n}C_{i}In(C_{i})$$

(3) $$\delta =100\times \sqrt{\sum_{i=1}^{n}C_{i}{\left(1-r_{i}/\bar{r}\right)}^{2}}$$

(4) $$\mathrm{VEC}=\sum_{i=1}^{n}C_{i}{\left(\mathrm{VEC}\right)}_{i}$$

where $\Omega_{ij}=4\Delta H_{ij}^{\mathrm{mix}(\mathrm{call})}$; Ci, Cj were the relative contents of different elements; ri was the atomic radius; $\bar{r}$ was the average radius; (VEC)_i_ was the valence electron concentration. Combined with the main formation conditions of the solid solution [18,29,30], ((i) −10 kJ/mol ≤ ΔHmix ≤ 5 kJ/mol; (ii) 12 J/k·mol ≤ ΔSmix ≤ 17.5 J/k·mol; (iii) 0 < δ < 8.5) and the VEC value, it was determined that point 3 and point 4 were both face-centered cubic structures. Point 3 was the Fe-Co-Cr phase, and point 4 was the Cu-rich phase (Cu-Al-Ni) [31].

**Figure 8:**
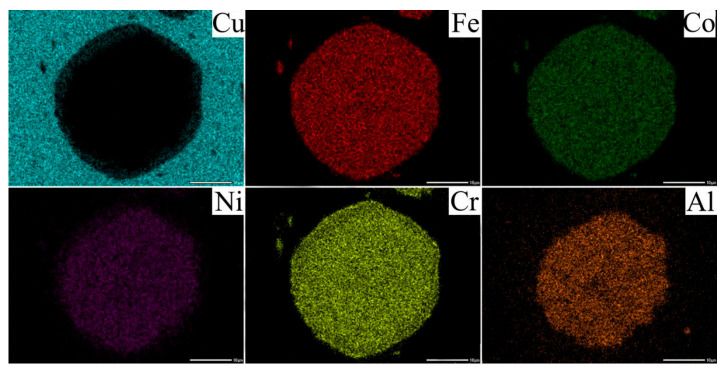
Surface scanning results of FSPed Cu@HEA/Cu-DL.

**Figure 9:**
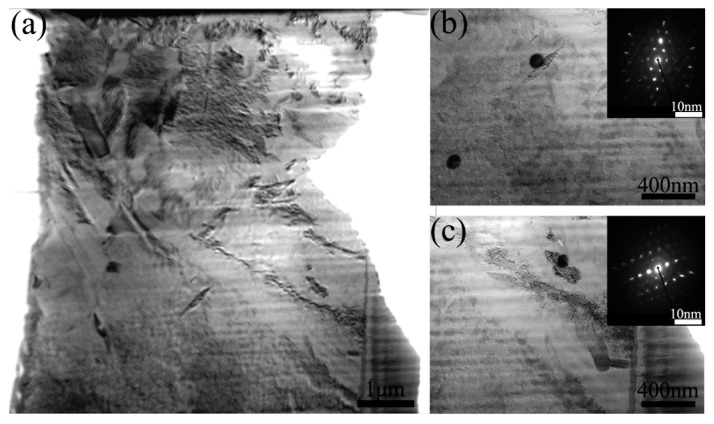
The TEM image of the HEA-Cu interface region of the FSPed Cu@HEA/Cu-DL. (**a**) the TEM image; (**b**,**c**) selected area electron diffraction.

The mapping results of the composite were shown in Figure 8. There was significant mutual diffusion among the elements at the interface of composite materials, leading to the formation of a distinct diffusion layer. This interdiffusion was predominantly facilitated by Cu, Al, and Ni elements. The distribution of each element was uniform, with no obvious segregation observed and no significant intermetallic compound formed. Figure 9 shows the TEM image of the diffusion layer. As shown in Figure 9b,c, the diffusion layer exhibited an FCC structure. It corresponded to the generation of the Fe-Co-Cr phase and the Cu-Al-Ni phase analyzed above.

### 3.3. Microstructure of Composites

**Figure 10:**
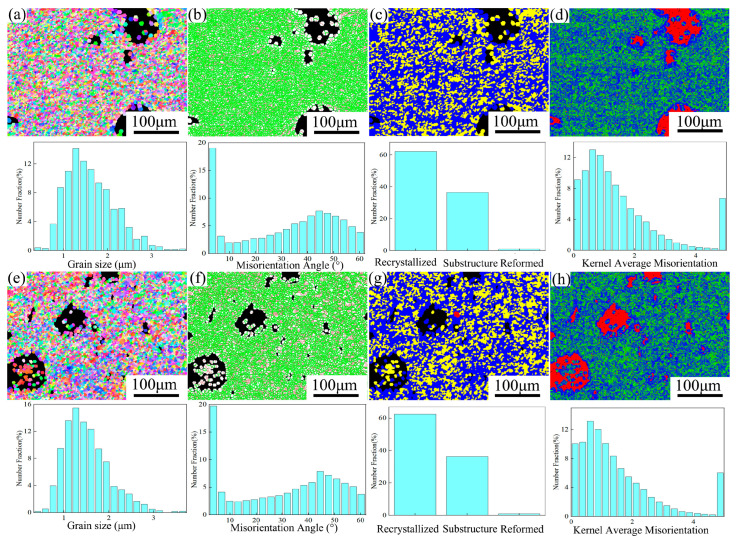
EBSD analysis results of composites. (**a**,**e**) grain size; (**b**,**f**) the HAGBs; (**c**,**g**) the recrystallization; (**d**,**h**) the KAM.

Figure 10 shows the EBSD analysis results of the composites. The average grain size of FSPed Cu@HEA/Cu was 1.5 μm, while that of FSPed Cu@HEA/Cu-DL was 1.46 μm, as illustrated in Figure 10a,e. The grain sizes of both composites were nearly identical to those of FSPed HEA/Cu and were finer than pure copper. In FSPed Cu@HEA/Cu, the proportion of HAGBs was 74.4%, and that of LAGBs was 25.6% (Figure 10b). In FSPed Cu@HEA/Cu-DL, the proportion of HAGBs was 72.7%, and that of LAGBs was 27.3% (Figure 10f). The proportion of recrystallized grains in both composites was the largest, about 62% (Figure 10c,g).

The reduction in grain size of the Cu matrix was primarily attributed to multi-pass FSP and the incorporation of HEA particles [16,18]. The formation of HAGBs and the alteration of grain structure were primarily attributed to dynamic recrystallization during FSP [32]. In this paper, electroless copper plating on the HEA particles increased the particle size slightly, which had little effect on the prepared composite. The FSP parameters of the two composites were the same. Therefore, the grain size, the proportion of HAGBs, and the proportion of recrystallized grains of the two composites were basically the same. Moreover, there was no significant difference compared with FSPed HEA/Cu (Table 1).

Figure 10d,h showed the KAM of the two composites. The KAM value in FSPed Cu@HEA/Cu was about 1.5, while the KAM value in FSPed Cu@HEA/Cu-DL was slightly lower, with a KAM value of about 1.3. For FSPed Cu@HEA/Cu, there was no obvious diffusion layer, and the intensity difference between the two sides of the interface was significant. High density dislocation was easily caused by the significant difference in strain rates at the interface. In the FSPed Cu@HEA/Cu-DL, the KAM value decreased as a result of the diffusion layer structure, which reduced the strain rate differential and the dislocation density between the two sides of the interface [18,24].

### 3.4. Texture

**Figure 11:**
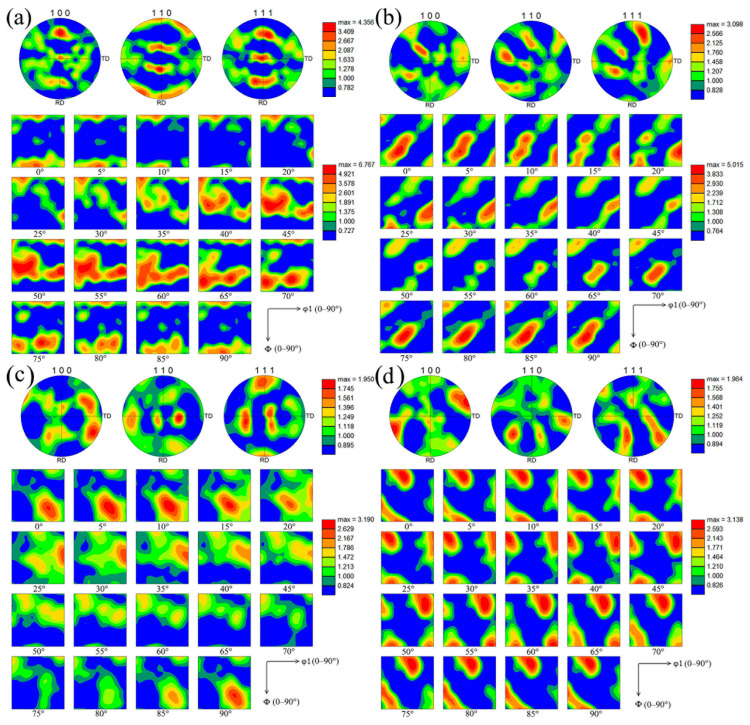
The pole figure and ODF diagram of samples: (**a**) Pure copper; (**b**) FSPed Cu; (**c**) FSPed HEA/Cu; (**d**) FSPed Cu@HEA/Cu-DL.

In order to more clearly study the texture evolution in the composite, the pole figure and Orientation Distribution Function graphs (ODF) of each sample were characterized in Figure 11. Figure 11a showed that the pole figures of {100}, {110}, and {111} in pure Cu all had high texture strength, and the texture orientation was symmetric along the RD direction, where the maximum texture strength was 4.35. The ODF results showed that the main texture types in pure Cu include {001}<110>CubeND texture, {112}<111>Copper texture, <001>||ND texture, <110>||ND texture, <111>||ND texture, <211>||ND, {223}<112> texture, {111} <112> texture and a small amount of {011}<211>Brass texture. Among them, {111} <112> had the highest texture strength, reaching 6.76. The <001>||ND texture, <110>||ND texture, <111>||ND texture, and <211>||ND were typical fiber textures, the formation of which was similar to the {011}<211>Brass texture, were related to the rolling process of materials.

As can be seen from Figure 11b, the texture orientation in FSPed Cu changed significantly. The texture strength in {100} and {110} pole figures was significantly reduced, while the {111} pole figure was still of high texture strength. The texture in the pole figure was completely asymmetric, which was caused by the uneven plastic deformation in all directions during FSP. In the shear texture component, there were high strength (111)[$\bar{1}$$\bar{1}$2]$A_{1}^{*}$ and (111)[11$\bar{2}$]$A_{2}^{*}$ textures, a small amount of (1$\bar{1}$1)[110]*A* and ($\bar{1}$1$\bar{1}$)[$\bar{1}$$\bar{1}$0]$\bar{A}$ textures, (1$\bar{1}$2)[110]*B* and ($\bar{1}$1$\bar{2}$)[$\bar{1}$$\bar{1}$0]$\bar{B}$ textures, and a small amount of {001}<101>*C* textures, indicating that there was high strain inside the metal [33]. The ODF diagram of FSPed Cu showed that its maximum texture strength was 5.0. In FSPed Cu, the fiber texture, {001}<110>CubeND texture, {111}<112> texture, {223}<112> texture disappeared completely, the {112}<111>Copper texture decreased significantly, and the {011}<211>Brass texture strength increased significantly. In addition, the {001}<100>Cube texture was formed. The presence of {011}<211>Brass texture and {001}<100>Cube texture indicated recrystallization of pure Cu after FSP (FSPed Cu). Due to the high strength of $A_{1}^{*}$ and $A_{2}^{*}$ textures, the degree of transition to *C* and *B* and textures was small, indicating that the recrystallization of FSPed Cu was not complete.

From Figure 11c, the strength of (1$\bar{1}$1)[110]*A* and ($\bar{1}$1$\bar{1}$)[$\bar{1}$$\bar{1}$0]$\bar{A}$ texture in FSPed HEA/Cu was the highest, and the strength of (1$\bar{1}$2)[110]*B* and ($\bar{1}$1$\bar{2}$)[$\bar{1}$$\bar{1}$0]$\bar{B}$ texture was increased compared with FSPed Cu. According to ODF, the strength of the {112}<111>Copper texture, {011}<211>Brass texture, and {001}<100>Cube texture in FSPed HEA/Cu decreased. The formation of recrystallization texture was also observed, including {001}<122>*P* texture, {013}<231>*Q* texture and {124}<211>*R* texture. The presence of these recrystallization textures and high-strength shear textures *B* and $\bar{B}$ indicates that the degree of recrystallization in FSPed HEA/Cu was further increased. Compared with pure Cu and FSPed Cu, the texture strength of FSPed HEA/Cu was significantly reduced. Khorrami [34] showed that the addition of reinforcement weakened the texture strength of the composites. This phenomenon was primarily attributed to grain boundary pinning by the reinforcement, which effectively suppressed grain growth. At the same time, the reinforcement distributed around the grain could be the nucleation site of the new grain recrystallization. The strength of the shear texture decreased in the composite, while that of the recrystallization texture increased. Studies have shown that the particle-stimulated nucleation mechanism made the material more likely to form random textures [35,36], thus reducing the overall texture strength of the material.

By comparison with Figure 11c,d, it can be seen that there were more (1$\bar{1}$2)[110]B, ($\bar{1}$1$\bar{2}$)[$\bar{1}$$\bar{1}$0]$\bar{B}$ textures and {001}<101>*C* textures in FSPed Cu@HEA/Cu-DL, while the intensity of (1$\bar{1}$1)[110]*A* and ($\bar{1}$1$\bar{1}$)[$\bar{1}$$\bar{1}$0]$\bar{A}$ textures decreased significantly. By observation of the ODF diagram, it was found that the {124}<211>*R* texture, {001}<100>Goss texture, {001}<122>*P* texture, {111}<112>*F* texture, and {001}<110>H texture with high intensity were generated in FSPed Cu@HEA/Cu-DL.The Cu@HEA-DL particles were more evenly distributed in the Cu matrix after FSP, which promoted recrystallization of the Cu matrix. This could be seen from the shear textures (*B*, $\bar{B}$, *C*), recrystallization textures (Goss, *P*, *R*, *F*, and *H*) generated in composites [37]. On the one hand, the increase in the degree of recrystallization was conducive to promoting the random orientation of the texture, resulting in a reduction in the overall texture strength. On the other hand, the uniform distribution of reinforced particles also led to an increase in strain strength, which enhanced the strength of the shear texture [38]. Finally, the overall texture strength of FSPed Cu@HEA/Cu-DL did not obviously change compared with FSPed HEA/Cu.

### 3.5. Mechanical Properties

**Figure 12:**
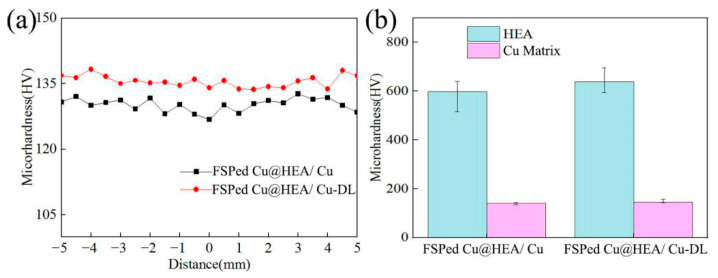
Microhardness test results of the composites. (**a**) surface microhardness; (**b**) average microhardness.

Figure 12 illustrates the microhardness of the composites. The microhardness of the Cu matrix was approximately 130 HV in the FSPed Cu@HEA/Cu, comparable to that of FSPed HEA/Cu. In contrast, the FSPed Cu@HEA/Cu-DL exhibited a higher matrix microhardness of 135 HV, representing a 60.3% increase over pure copper. Furthermore, the HEA particles in FSPed Cu@HEA/Cu-DL showed enhanced hardness, averaging 638.2 HV, compared to those in FSPed Cu@HEA/Cu. The uniform distribution of reinforcement exerted a considerable influence on the composite properties. The distribution curve of microhardness did not fluctuate considerably, indicating a homogeneous dispersion of the reinforcement within the Cu matrix, which was facilitated by the electroless copper plating layer on the HEA surfaces. This copper layer improved particle wettability and mitigated agglomeration during FSP, thereby enhancing distribution uniformity and composite strength [39]. However, the presence of electroless copper plating layer also slightly reduced the effective volume fraction of HEA reinforcement, which would typically diminish strength [40]. The two factors offset each other, resulting in no significant change in the matrix microhardness of the FSPed Cu@HEA/Cu. The further increase in microhardness of the Cu matrix in FSPed Cu@HEA/Cu-DL was attributed to the prior heat treatment of Cu@HEA. This treatment refined the grain structure and increased the microhardness of the HEA particles [41] (638.2 HV), resulting in an enhanced effect of load transfer strengthening.

**Figure 13:**
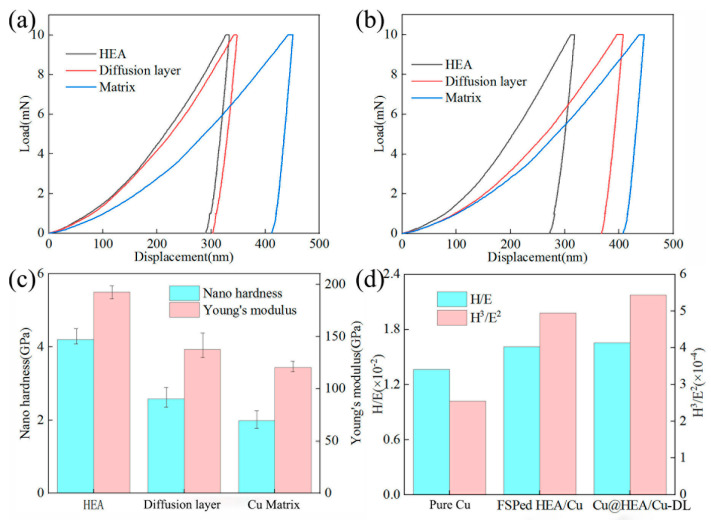
The nanoindentation test results of the composites (**a**) without, (**b**) with, (**c**) nanohardness—Young’s modulus, (**d**) H/E and H^3^/E^2^.

Figure 13 shows the nanoindentation test results of composites. In the FSPed Cu@HEA/Cu-DL, the nano-hardness of HEA particles, diffusion layer, and Cu matrix were 4.2 GPa, 2.58 GPa, 1.99 GPa, and the elastic modulus were 192.54 GPa, 137.17 GPa, and 120.43 GPa, respectively. Figure 13d showed the ratio of nano-hardness to elastic modulus (H/E, H^3^/E^2^) of Cu matrix in pure Cu, FSPed HEA/Cu, and FSPed Cu@HEA/Cu-DL. The smaller the ratio of nano-hardness to elastic modulus, the smaller the elastic recovery of indentation, and the deeper the residual indentation after unloading. Compared with pure Cu and FSPed HEA/Cu, FSPed Cu@HEA/Cu-DL exhibited a higher ratio of H/E to H^3^/E^2^. This result demonstrates that incorporating Cu@HEA-DL particles effectively enhanced the resistance of the Cu matrix to both elastic strain failure and plastic deformation.

**Figure 14:**
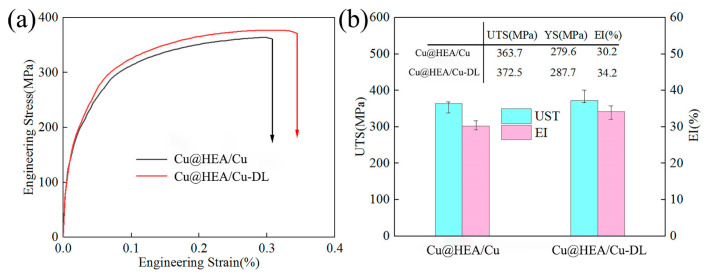
Tensile test results of the composites (**a**) Stress-strain curve; (**b**) Tensile result.

Figure 14 shows the tensile test results of the composites. The FSPed Cu@HEA/Cu exhibited tensile strength, yield strength, and elongation values of 363.7 MPa, 279.6 MPa, and 30.2%, respectively, comparable to those of FSPed HEA/Cu. In comparison, the FSPed Cu@HEA/Cu-DL demonstrated superior tensile properties, with corresponding values of 372.5 MPa, 287.7 MPa, and 34.2%. These represent an increased of 20.4% in tensile strength and 54% in elongation over pure copper. The results showed that FSPed Cu@HEA/Cu-DL enhanced mechanical properties relative to both FSPed HEA/Cu and FSPed Cu@HEA/Cu. The results showed that the mechanical properties of FSPed Cu@HEA/Cu-DL were enhanced relative to both FSPed HEA/Cu and FSPed Cu@HEA/Cu.

**Figure 15:**
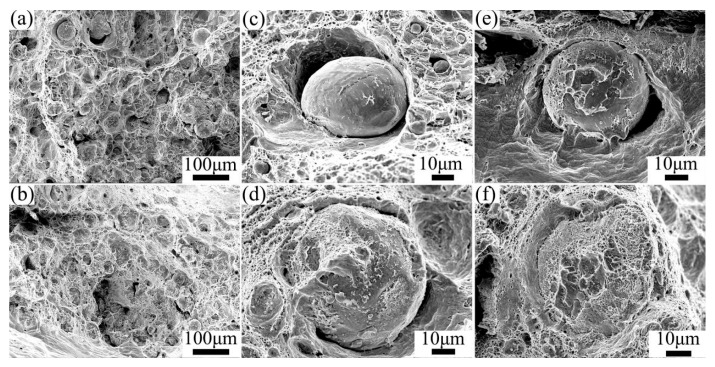
Tensile fracture morphology of composites (**a**,**c**,**e**) FSPed Cu@HEA/Cu; (**b**,**d**,**f**) FSPed Cu@HEA/Cu-DL.

Figure 15 shows the tensile fracture morphology of each sample. Abundant dimples on the Cu matrix in both composites (Figure 15a,b) indicated a predominant ductile fracture mode. The interfacial fracture behavior was shown in Figure 15c,e. In FSPed Cu@HEA/Cu, the surface of HEA particles was mostly smooth, exhibiting the characteristics of brittle fracture. There were dimples and tearing edges in a few areas, which showed the characteristics of ductile fracture. This was the reason for the plasticity of the composites reinforced by HEA particles over conventional ceramic particles. The FSPed Cu@HEA/Cu-DL showed extensive dimples and tear ridges on the HEA particle surfaces (Figure 15d), confirming a fully ductile fracture mechanism. Some fractured HEA particles also displayed dimpled rupture surfaces (Figure 15f), further affirming enhanced interfacial bonding and ductility.

**Figure 16:**
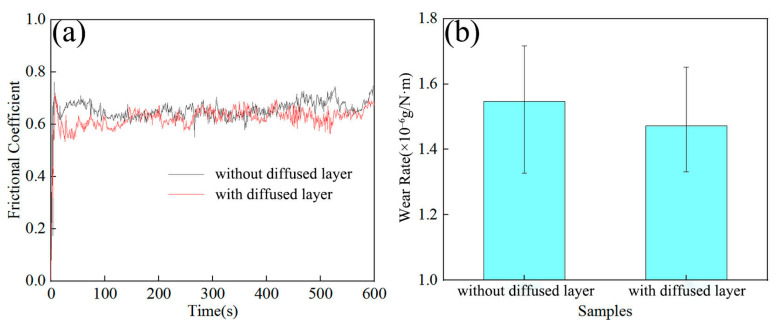
Test results of friction and wear properties of composites: (**a**) Friction coefficient; (**b**) wear rate.

Figure 16 presents the friction and wear properties of the composites. The FSPed Cu@HEA/Cu exhibited a friction coefficient of 0.657 and a wear rate of 1.547 × 10^−6^ g/N·m, values slightly lower than those of FSPed HEA/Cu. The FSPed Cu@HEA/Cu-DL showed further improved tribological performance, with a friction coefficient of 0.642 and a wear rate of 1.472 × 10^−6^ g/N·m, representing a 33.7% reduction compared to pure copper. The friction coefficient curve fluctuated gently, particularly for FSPed Cu@HEA/Cu-DL, suggesting that the interfacial diffusion layer contributed to enhanced tribological behavior. These results confirmed the superior wear resistance of the FSPed Cu@HEA/Cu-DL.

**Figure 17:**
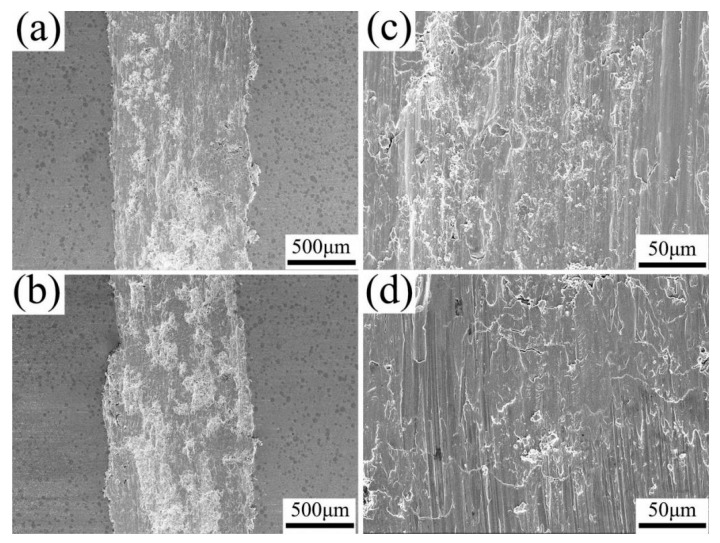
Wear morphology of composites (**a**,**c**) FSPed Cu@HEA/Cu; (**b**,**d**) FSPed Cu@HEA/Cu-DL.

Figure 17 shows the wear morphology of the composite. The worn surface of FSPed Cu@HEA/Cu appeared relatively smooth, with minor debris and furrows, exhibiting characteristics indicative of abrasive wear (Figure 17a,b). According to Figure 17c,d, the FSPed Cu@HEA/Cu-DL displayed an even smoother surface with shallower furrows, demonstrating superior wear resistance. Notably, nearly no HEA particles were removed from the Cu matrix, which can be attributed to the interfacial diffusion layer formed between HEA and the Cu matrix. This structure played a critical role in enhancing the wear performance of the composite.

## 4. Discussion

### 4.1. Interface Structure

**Figure 18:**
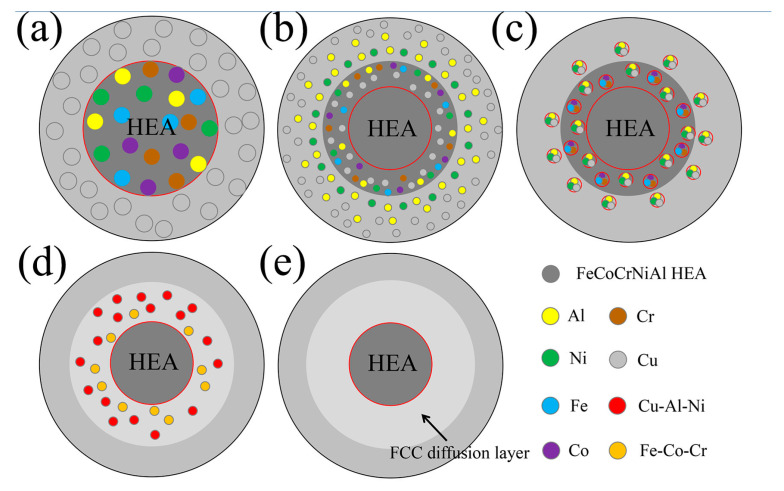
Interface diffusion diagram of HEA particles and Cu matrix.(**a**–**e**) Interface diffusion process.

The interface diffusion diagram was displayed in Figure 18. As depicted in Figure 18a, the initial Cu@HEA particle exhibited a sharp interface, with Fe, Co, Ni, Cr, and Al uniformly distributed in the HEA particle. Following heat treatment, elemental interdiffusion occurred across the interface (Figure 18b). Al and Ni from the HEA diffused into the Cu layer, while Cu migrated toward the HEA periphery but did not penetrate the HEA core due to the limited diffusion time. The out-diffusion of Al and Ni induced elemental segregation of Fe, Co, and Cr at the edge of HEA particles. At the same time, the Al and Ni elements that diffused into the Cu layer were bound to Cu elements, and the Cu elements that diffused into HEA were bound to the small amount of Al and Ni elements (Figure 18c). Subsequently, Cu-Al-Ni and Fe-Co-Cr phases were formed at the interface (Figure 18d). These phases eventually developed into a continuous FCC-structured diffusion layer (Figure 18e).

### 4.2. Strengthening Mechanism

The strengthening mechanisms of particle-reinforced MMCs primarily include fine-grain strengthening, Orowan strengthening, load transfer strengthening, and dislocation strengthening. In this paper, these mechanisms operated synergistically during FSP, collectively enhancing the overall mechanical properties of the composite. It was essential to investigate the specific contributions of each strengthening mechanism. The following would be the analysis of these strengthening mechanisms.

(1) According to the Hall-Petch [42] equation:
  (5) $$\sigma_{s}=\sigma_{\mathrm{sc}}+kd^{-1/2}$$
  where σ_sc_ was the yield strength of a single crystal. The yield strength of the material (σ_s_) was inversely proportional to the average grain size (d). Based on equation (5), the yield strength increment due to fine-grain strengthening was given by [43]:
  (6) $$\Delta \sigma_{G}=k\cdot \left(d^{-1/2}-d_{0}^{-1/2}\right)$$
  where d_0_ and d represented the average grain sizes of pure copper and the composite, respectively. k was a constant taken as 4.5 MPa·mm1/2 [44]. The calculated Δσ_G_ was 41.2 MPa.
(2) The increment of yield strength caused by Orowan strengthening can be expressed as [45,46]:
  (7) $$\Delta \sigma_{\mathrm{Orowan}}=\frac{\eta M}{2\pi \sqrt{1-v}}\frac{G_{m}b}{L}\ln\frac{d_{p}}{2b}$$
  (8) $$G_{m}=\frac{E}{2\left(1+v\right)}$$
  L = d_p_[(1/2V_p_)^1/3^−1] (9)
  where η was a constant of 0.85 related to the particle distribution state; M was the Taylor factor, taken as 3.06 for the FCC metal matrix; ν was Poisson’s ratio, equal to 0.34; Gm represented the shear modulus of the matrix; b denoted the Burgers vector, with a value of 0.255 [44]; L correspond to the interparticle spacing; and E was the elastic modulus of the metal matrix. According to the above equation, in theory, the higher the volume fraction of reinforcement, the more obvious the effect of Orowan strengthening. But in fact, when the reinforcement volume fraction was high, the dislocation was difficult to slip and it was difficult to circumnavigate through the particles. Therefore, Orowan strengthening was more obvious in the lower volume fraction range. When the size of reinforcement was large, the spacing of adjacent particles increased. When the dislocation bypassed the particle, the effective dislocation ring could not be formed, and the Orowan strengthening effect was not significant. The calculated Δσ_Orowan_ was 0.3 MPa.
(3) The increment of yield strength caused by dislocation strengthening can be expressed as [47]:
  (10) $$\Delta \sigma_{\mathrm{CTE}}=KG_{m}b\sqrt{\rho_{\mathrm{CTE}}}$$
  (11) $$\rho_{\mathrm{CTE}}=\frac{BV_{P}\cdot \Delta \mathrm{CET}\cdot \Delta T}{bd_{p}\left(1-V_{p}\right)}$$
  where K was a constant with a value of 1.25, ρ_CTE_ denoted the dislocation density induced by the difference in the coefficient of thermal expansion (CTE), ΔCTE represented the CTE mismatch between the reinforcement and the Cu matrix, ΔT was the temperature difference between the composite preparation temperature and room temperature, and B was a geometric constant taken as 12. According to the equation, the calculated Δσ_CTE_ was 12.7 MPa.
(4) The increment of yield strength caused by load transfer strengthening was expressed as follows [48]:
  (12) $$\Delta \sigma_{\mathrm{Load}}=V_{p}\sigma_{0}\frac{\left(1+t\right)A}{4l}$$
  where σ_0_ was the yield strength of pure Cu, l and t were the lengths of the reinforcement perpendicular and parallel to the load direction, respectively, and A was the length-diameter ratio of the reinforcement. For equiaxial particles, Δσ_Load_ can be expressed as [49]:
  (13) $$\Delta \sigma_{\mathrm{Load}}=0.5V_{p}\sigma_{0}$$
  as indicated by the equation, the calculated Δσ_Load_ was 15.7 MPa.

**Figure 19:**
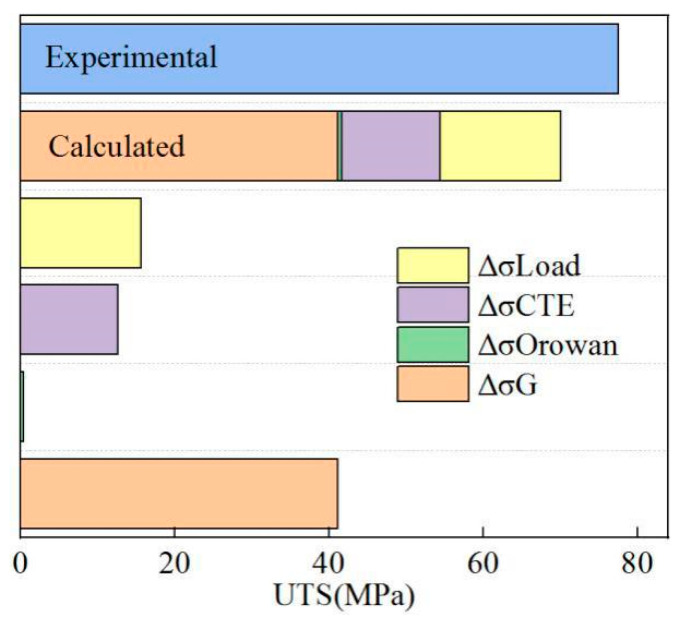
Comparison between calculated yield strength and actual yield strength.

Figure 19 compares the theoretical and experimentally measured increments of yield strength. The theoretical calculation was 66.9 MPa, which was 7.6 MPa lower than the measured value of 77.5 MPa. This result was related to the theoretical calculation value of Orowan strengthening. The calculation incorporated the average size of the initial HEA particles (about 28 μm). However, FSP led to particle fragmentation, producing submicron particles (<1 μm) that significantly enhanced Orowan strengthening [25]. Furthermore, the narrow interfacial diffusion layer may promote stress concentration during the tensile process, adversely affecting composite properties. Increasing the diffusion layer width can strengthen the HEA–matrix interface, mitigate stress concentration, suppress crack initiation, and improve overall ductility.

**Figure 20:**
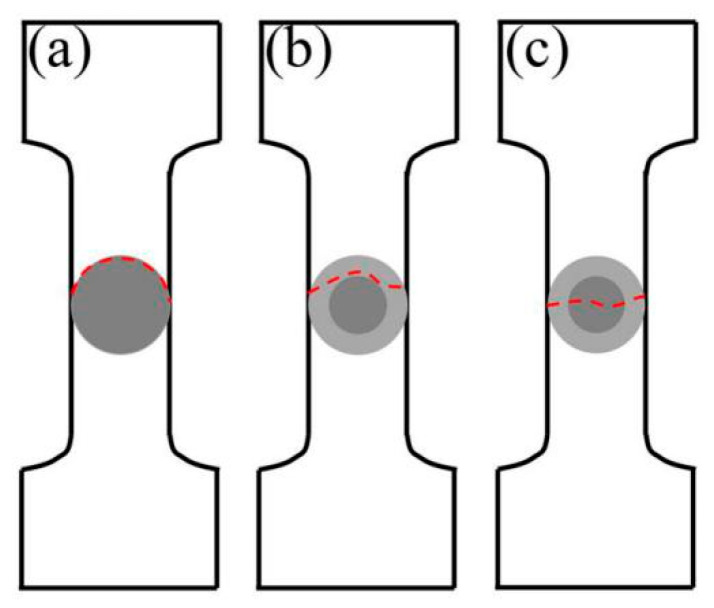
Diagram of fracture patterns. (**a**) interface fracture; (**b**) the diffusion layer fracture; (**c**) the HEA particles fracture.

Figure 20 shows the fracture pattern of the HEA/Cu interface in composites. In FSPed Cu@HEA/Cu, where no distinct diffusion layer formed, fracture occurred along the interface under tensile loading (Figure 20a). In contrast, the fracture in FSPed Cu@HEA/Cu-DL took place within the diffusion layer (Figure 20b). As indicated by earlier EDS analysis, this layer consisted mainly of a Cu-rich phase and an Fe-Co-Ni phase, both with an FCC structure that offered improved plasticity over the BCC structure. After FSP, the texture strength of the composite decreases (Figure 11). Therefore, the elongation of FSPed Cu@HEA/Cu-DL was improved. A small number of fractures occurred on HEA particles, as shown in Figure 20c. HEA particles with the BCC structure have relatively high strength. However, small HEA particles were affected by element diffusion and transformed from BCC structure to FCC structure, resulting in a decrease in hardness and leading to fracture. From Figure 15, the broken HEA particles were mainly small ones.

**Figure 21:**
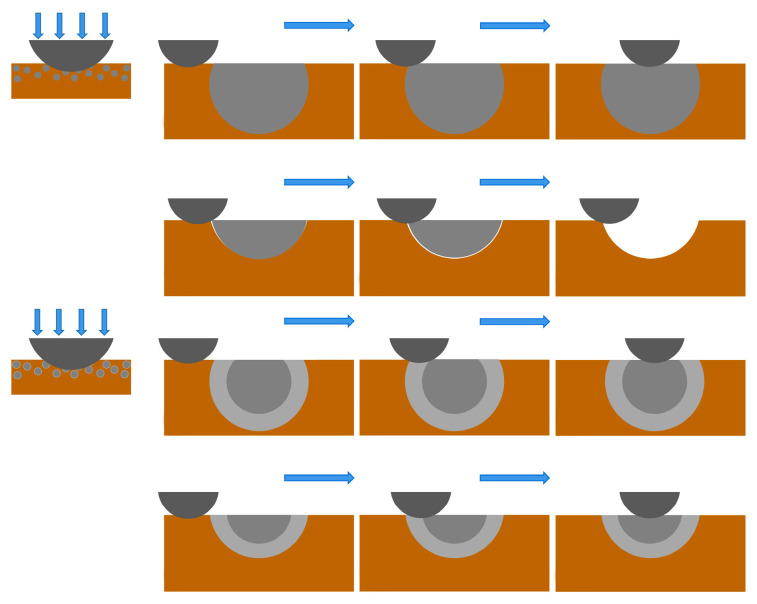
Schematic of the wear mechanism.

Figure 21 shows the wear mechanism diagram of the composites. For FSPed Cu@HEA/Cu, when the steel ball was in contact with the composite under vertical load and sliding friction at a certain speed, the Cu matrix with low hardness inevitably fell off from the composite and formed a furrow, while HEA particles with higher hardness can effectively resist the applied load, thereby reducing direct contact between the steel balls and the Cu matrix, and consequently enhancing the overall wear resistance of the composite. However, with the prolongation of wear time, the binding ability decreased, thereby increasing the likelihood of HEA particle detachment from the matrix, which ultimately led to a significant reduction in the wear resistance of the composite. For FSPed Cu@HEA/Cu-DL, the steel ball needed to pass through a diffusion layer when sliding from the Cu matrix to the HEA particles. The diffusion layer played a transitional role during the friction and wear process, reducing the friction force generated as the steel ball moved directly from the Cu matrix to the HEA, contributing to a decrease in the friction coefficient. At the same time, the hardness of HEA particles was increased after heat treatment, which further strengthens the load transfer effect. The combined effect of both factors contributed to the enhancement of the wear resistance. In addition, the diffusion layer structure greatly improved the interface bonding ability between HEA particles and Cu matrix. It can be expected that even after a longer period of wear, it can maintain good adhesion, ensuring that HEA particles provide continuous load resistance and maintain long-term wear resistance.

## 5. Conclusions

In this paper, HEA particles were initially subjected to electroless copper plating followed by heat treatment to produce Cu@HEA-DL particles with an interfacial diffusion layer. These particles were incorporated into a copper matrix via FSP to fabricate composites. The research results on the composite were as follows:

(1) Interdiffusion of elements occurred at the interface: Al and Ni from the HEA diffused into the Cu matrix, while Cu diffused into the HEA, resulting in the formation of an FCC-structured diffusion layer approximately 3.5 μm. The layer primarily consisted of Cu-Al-Ni phase and Fe-Co-Cr phase.
(2) The composite exhibited an average grain size of 1.46 μm and a maximum texture strength of 3.13. Recrystallization textures, such as Goss and P textures, were identified.
(3) The composite showed an average microhardness of 135 HV, with tensile strength and elongation reaching 372.5 MPa and 34.2%, respectively. The fracture morphology shows a typical ductile fracture. The friction coefficient and wear rate of the composite were 0.642 and 1.472 × 10−6 g/N·m, respectively.
(4) The contributions to the overall strength were quantified as follows: grain refinement strengthening accounted for 41.2 MPa, Orowan strengthening for 0.3 MPa, dislocation strengthening for 12.7 MPa, and load transfer strengthening for 15.7 MPa.

This paper demonstrated that by pre-treating the high-entropy alloy particles and then preparing the composite, the mechanical properties of the materials could be enhanced. However, under the given heat treatment process parameters, the improvement in the properties of the composite was limited. Therefore, in the subsequent research, we will investigate the effects of different heat treatment process parameters on the mechanical properties of the composite.

## Figures and Tables

**Table 1:** The mass fraction of each element in HEA particle.

Element	Fe	Co	Ni	Cr	Al
Content (%)	22.23	23.53	22.79	20.62	10.83

**Table 2:** Main parameters of the material.

	Pure Cu	FSPed HEA/Cu
Grain size (μm)	18.2	1.6
High-angle grain boundaries (HAGBs)	54.3%	74.9
Low-angle grain boundaries (LAGBs)	45.7	25.1
Recrystallized grains	26%	62%
Kernal average misorientation (KAM)	1.061	1.501
Microhardness (HV)	84.2	130.4
Nanohardness (GPa)	1.37	1.91
Modulus of elasticity (GPa)	100.43	118.76
Tensile strength (MPa)	309	362
Elongation (%)	22.2	30.6
Friction coefficient	0.753	0.655
Wear rate (×10^−6^ g/N·m)	2.23	1.57

**Table 3:** Composition of plating solution and reaction conditions.

Materials/Test Conditions	CuSO_4_·5H_2_O	KNaC_4_H_4_O_6_·5H_2_O	NaOH	HCHO	Temperature
Content	70 g/L	170 g/L	50 g/L	50 g/L	50 °C

**Table 4:** The EDS analysis results of each point in Figure 7d.

	Cu	Fe	Co	Ni	Cr	Al
1	--	22.76	22.44	22.68	21.25	10.87
2	--	23.72	24.17	22.69	19.69	9.73
3	11.02	24.98	25.43	14.33	21.88	2.36
4	31.16	19.22	18.63	13.43	15.29	2.27

## Data Availability

The original contributions presented in this study are included in the article. Further inquiries can be directed to the corresponding author.
